# Rayleigh–Schrödinger Perturbation Theory and Nonadditive Thermodynamics

**DOI:** 10.1021/acs.jpcb.3c01525

**Published:** 2023-05-25

**Authors:** Rodrigo de Miguel

**Affiliations:** Norwegian University of Science and Technology, 7491 Trondheim, Norway

## Abstract

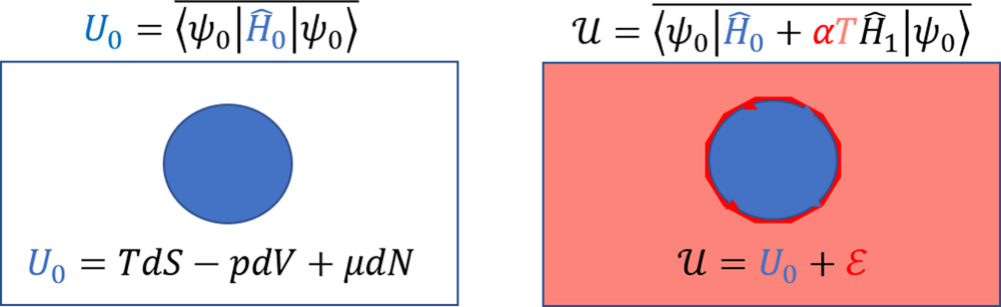

Physical chemists reconcile the empirical theory of classical thermodynamics with the quantum nature of matter and energy when they recover thermodynamics from a statistical mechanical treatment of the individual particles’ quantized eigenspectrum. The conclusion is that, when systems are very large collections of particles, interactions between adjacent systems are comparatively negligible, resulting in an additive thermodynamic framework where the energy of a composite system *AB* may be expressed as the sum of the individual energies of subsystems *A* and *B*. This powerful theory is consistent with quantum theory, and it accurately describes the macroscopic properties of sufficiently large systems subject to comparatively short-ranged interactions. Nevertheless, classical thermodynamics has its limitations. Its main drawback is the theory’s failure to accurately describe systems not sufficiently large for the aforementioned interaction to be neglected. This shortcoming was addressed by the celebrated chemist Terrell L. Hill in the 1960s when he generalized classical thermodynamics by adding a phenomenological energy term to describe systems not captured by the additivity ansatz (i.e., *AB* ≠ *A* + *B*) of classical thermodynamics. Despite its elegance and success, Hill’s generalization mostly remained a specialist tool rather than becoming part of the standard chemical thermodynamics corpus. A probable reason is that, in contrast to the classical large-system case, Hill’s small-system framework does not reconcile with a thermostatistical treatment of quantum mechanical eigenenergies. In this work we show that, by introducing a temperature-dependent perturbation in the particles’ energy spectrum, Hill’s generalized framework is in fact recovered with a simple thermostatistical analysis accessible to physical chemists.

## 1 Introduction

Classical thermodynamics considers very large systems subject to short-ranged interactions. In this situation, it is safe to neglect interaction energies between adjacent subsystems and simply express the energy of a composite system *AB* as the sum of the individual energies of subsystems *A* and *B*. This additive thermodynamic framework is a very powerful tool for the prediction of the collective properties of large collections of particles.

At the molecular level, we reconcile thermodynamics with the quantum nature of matter and energy by confirming that the thermodynamic framework, with collective properties such as temperature, pressure, and chemical potential, can indeed be obtained from a statistical mechanical treatment of the individual particle’s quantum energies (see, e.g., ref (1)).

Nevertheless, classical thermodynamics has its limitations. Its main drawback is the theory’s inability to accurately describe systems not sufficiently large for their interaction with the environment to be negligible. This problem is not limited to quantum systems, for what makes a system *small* is not so much its sheer size as how its size compares to the range of the interactions affecting the system. From this point of view, a solar system may be thought of as *small*: two adjacent and identical solar systems would not have twice the energy as one solar system.^2^ Yet a water droplet may well be thought of as *large*.^3^ This issue was addressed by the celebrated physical chemist Terrel L. Hill in the early 1960s, when he generalized classical thermodynamics to describe *small* systems.^4,5^ Hill considered a homogeneous macroscopic system at equilibrium and abstractly subdivided it into very small subsystems. Due to the subsystems’ small size, interaction energies between them are comparatively significant, and the usual energy additivity no longer applies. This creates the need for an additional phenomenological term, a *subdivision potential* that accounts for the interaction between a small subsystem and its surroundings. Far from being a mere curiosity, Hill’s small-system method (later termed nanothermodynamics^6−8^) has found applications in different domains, such as transport in porous media,^9^ drug delivery,^10^ and materials science.^11^

Yet, when it comes to undergraduate thermodynamics, Hill’s generalized theory has a distinct disadvantage with respect to classical extensive thermodynamics. Hill’s framework, with its additional energy term, does not simply emerge from a thermostatistical treatment of quantum eigenergies. The usual statistical bridge between the quantum and the thermodynamic description appears to be missing. This may be a reason why Hill’s otherwise intuitive phenomenological theory remained a specialist tool rather than becoming part of the standard physical chemistry corpus. In this work, we show that Hill’s framework may indeed be recovered by introducing a simple thermal perturbation in the system’s eigenenergies. The derivation is accessible to all physical chemists familiar with Rayleigh–Schrödinger perturbation theory^12^ and elementary statistical thermodynamics,^1^ and it may serve to give the wide physical chemistry community molecular insight into a generalized thermodynamic theory shown to have a wide range of applicability.

The remainder of this paper is organized as follows. We start with a short summary of Hill’s generalized thermodynamic theory. We then propose a perturbation theory and a thermostatistical treatment that results in Hill’s theory and an extended second law of thermodynamics. We then consider a perturbed harmonic oscillator as a simple yet illustrative example. We close with some concluding remarks.

## 2 Hill’s Thermodynamics

In the following we provide a brief and modest summary of Hill’s generalized thermodynamic theory.^5^ Hill starts by considering a homogeneous macroscopic system at equilibrium temperature *T*, pressure *p*, and chemical potential μ. If this system were thought of as consisting of two macroscopic subsystems, the energy of interaction between these two subsystems would be negligible compared to each of the energies. If the two subsystems were identical, each of them would have *exactly* half of the total energy, and the same *T*, *p*, and μ as the original system.

However, if the macroscopic system were divided into many much smaller subsystems, the internal energy of each subsystem would become comparable to the interaction energy between them. In contrast to the macroscopic additive case, the interaction energy can no longer be neglected, and the subsystem’s internal energy  is given by
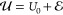
1

The first term in (1) is given by the classical Euler equation
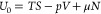
2where *S*, *V*, and *N* are, respectively, the subsystem’s entropy, volume, and number of particles. The additional term  is known as the *subdivision potential*, i.e., the energy contribution into a subsystem that results from interaction with other subsystems. It may be thought of as the difference between a subsystem’s true internal energy  and the extensive expression *U*_0_. For very large subsystems (the classical case), the subdivision potential  becomes negligible and the standard thermodynamic theory is recovered.

However, due to the presence of the subdivision potential  in (1), the internal energy  of the small system ceases to be a linear homogeneous function of *S*, *V*, and *N*. This additional term is the cornerstone of Hill’s nanothermodynamics. As Hill wrote, *small system thermodynamics departs from macroscopic thermodynamics in that**is not a linear homogeneous function of [S*, *V*, *and N]*. *Hence an extra term occurs in [(1)]*. *These last two sentences epitomize the whole book* (ref (5), p 24).

### 2.1 Environmental Constraints

If we were describing a small open system with a definite chemical potential and temperature (imposed by the heat and particle reservoir surrounding the system), then the additional energy term  in (1) can only stem from alterations in pressure. Interactions with the environment cause the system’s pressure to depart from *p* and become instead an effective *p̂*. Then, the deviation in the system’s internal energy is given by (ref (5), pp 10, 24)
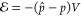
3

If the small system of interest were, instead, a closed system with a definite pressure and temperature, then any additional energy term must result from an alteration in chemical potential. The presence of the environment causes the system’s chemical potential to deviate from μ and become instead an effective μ̂. The correction to the system’s internal energy is then given by (ref (5), pp 16, 24)
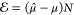
4

If the only environmental constraint were the temperature, interactions with the environment would cause deviations both in pressure and chemical potential. As a result, the energy correction  becomes (ref (5), p. 24)
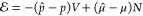
5

In the macroscopic limit, the internal energy of the system becomes much larger than the energy resulting from its interactions with the environment. As a result  regardless of the system’s environmental constraints. In other words: ensemble equivalence is recovered in the thermodynamic limit.

## 3 Thermally Induced Spectrum Perturbations

In the following, we show how Hill’s thermodynamic theory summarized above results from a standard thermostatistical treatment of energy levels if these are perturbed by the temperature of the heat bath.

We consider a simple system embedded in a heat bath. The total Hamiltonian  for the system-bath complex is given by
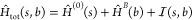
6where  is the Hamiltonian of the bare system in state *s*,  is the Hamiltonian of the bath in state *b*, and  is the interaction between the system and the heat bath.

In recent years, a *statistical mechanics and thermodynamics at strong coupling* has been developed on the basis of a *Hamiltonian of mean force*.^13−16^ Averaging  and  over the environment  results in a temperature-dependent Hamiltonian of mean force *Ĥ* for the system given by^13^
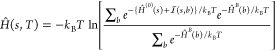
7where *k*_B_ is Boltzmann’s constant and *T* is the heat bath’s tempeature. If the interactions  with the environment are negligible compared to the bare system’s Hamiltonian, the Hamiltonian of mean force *Ĥ* reduces to the bare (and temperature-independent) .

In other words, the coupling between the system and the environment perturbs the system’s energy landscape. If the coupling is weak, then the change in the original eigenenergies is negligible. On the other hand, when the coupling is strong compared to the system’s own energy, the spectrum of mean force changes significantly. In general, the spectrum is modified by the energy exchange, which is regulated by the heat bath’s temperature *T*. As a result, the Hamiltonian of mean force *Ĥ* becomes dependent on the external temperature and may be written as as a power series in *T*
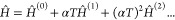
8where α is a positive parameter modeling the strength of the coupling between the particular system and the specific heat bath. Likewise, the eigenstates may be written as
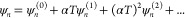
9as can the corresponding eigenenergies
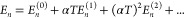
10where the ψ_*n*_^(0)^ and *E*_*n*_^(0)^ are, respectively, the eigenfunctions and eigenvalues of the unperturbed purely mechanical Hamiltonian .

If the perturbed state is sufficiently similar to the unperturbed state (wave function), we may safely neglect higher-order perturbations and apply standard first-order Rayleigh–Schrödinger perturbation theory (see, e.g., ref (12)). We then obtain a corrected energy given by
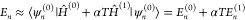
11

In the absence of thermal perturbations, the temperature-dependent energies reduce to the usual temperature-independent, purely mechanical energies. In general, however, temperature dependence does emerge into effective energy levels.^17^ Indeed, temperature-dependent perturbations of energy levels have long been observed, for example, in semiconductors,^18^ and they play an important role in the properties of modern nanoscaled materials (see, e.g., ref (19)).

### 3.1 Generalized Thermostatistics

We consider now an ensemble of systems and write the partition function  with the—now temperature-dependent—perturbed energies (11) in the Boltzmann factor.
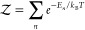
12

Applying the standard machinery of statistical thermodynamics,^1^ we may obtain the energy of the system from the partition function as , resulting in
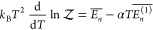
13where the overbar denotes the average over all available microstates *n*. This generalized expression departs from the usual expression by the presence of the last term, which results from the temperature-dependent perturbation in the system’s Hamiltonian. From (13) and (11) we may conclude that the averaged zeroth-order energy is given by the usual expression
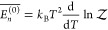
14

With a modest amount of foresight, we may now look at (1) and identify the system’s perturbed energy  as , the zeroth-order energy  as *U*_0_, and the perturbation  as the excess energy  resulting from the strong interactions with the environment. When the thermal perturbation is absent (isolated system) or negligible (large system), expressions (8)–(13) all simplify to their familiar form.

The perturbed system’s pressure *p̂* is given by  and it deviates from the pressure *p* = –*∂U*_0_/*∂V* the system would have in the absence of interactions. The pressure difference due to the perturbation is easily shown to be given by
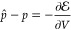
15Likewise, the perturbed system’s chemical potential μ̂ is given by  and it differs from the chemical potential μ = *∂U*_0_/*∂N* of the bare system by
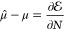
16

From (15) and (16), we conclude that the environmental perturbations change the internal energy of the system by an amount  given by expressions (3)–(5), which were originally introduced using purely thermodynamic arguments. In support of Hill’s purely thermodynamic approach, the subdivision potential has a thermostatistical basis in terms of a thermally perturbed effective Hamiltonian (8).

The idea of a statistical mechanics allowing for temperature-dependent energy levels is far from new. It was first proposed by Rushbrooke^20^ and later refined by Elcock and Landsberg in the late 1950s.^17^ More recently, this idea has resurfaced in a framework known as *statistical mechanics at strong coupling*.^13,21^ Incidentlly, the framework of temperature-dependent energy levels is acknowledged in Pathria’s landmark tektbook in statistical mechanics (see ref (22), chapter 3, footnote 1).

### 3.2 Extended Second Law

The temperature *T* of the system-bath complex is given by the usual equilibrium expression
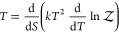
17which, using (13) and (11) becomes
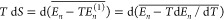
18or
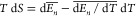
19Like (13), this expression departs from the usual equilibrium expression by an additional term that accounts for the possible temperature dependence of the energy levels. The process described by (19) is the following: When a system comes into thermal contact with a heat bath, it absorbs an amount of heat . This heat excites the system up its energy/entropy landscape, increasing its temperature by *dT*. The system then cools back down toward the heat bath temperature by doing work  on the environment. This work is performed in an an isentropic process where the system widens its potential energy surface, changing the energy levels without changing the populations. Then, the eigenstates’ contribution to the system’s energy changes from the original  to the corrected , and the effective heat received by the system is thereby reduced from  to a smaller .^23^ Indeed, it has been shown that two distinct types of temperature dependence emerge from the strong coupling between the system and the heat bath: While the *E*_*n*_ appearing in the Boltzmann factor in (12) determine the probability that a state is occupied, the (*E*_*n*_ – *TdE*_*n*_/*dT*) in (18) determines the contribution made to the total energy by that state *when it is occupied*.^24,25^

Expression (19) is an extended second law of thermodynamics, originally proposed by Shental and Kanter in the context of information theory.^26^ This extended law has been successfully invoked to describe irreversible processes in themoelectrical devices,^27,28^ model optomechanical oscillators,^29,30^ and to provide a simple thermostatistical description of the somewhat mystifying thermophilic motion exhibited by some macromolecules such as proteins.^25^ The usual second law is recovered in the limit where the thermal perturbation of the spectrum is absent (or negligible) and the last term in (19) vanishes.

## 4 Example: Harmonic Oscillator

A heat bath may be modeled as a large collection of oscillators (see, e.g., refs (31 & 32)). For illustrative simplicity, we shall focus on just one of these oscillators and model it as a harmonic oscillator embedded in a heat bath made up of all the other oscillators. The unperturbed potential and energy spectrum of the system of interest are, respectively, given by
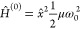
20
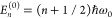
21where *x̂* is the position operator, *ℏ* is the reduced Planck constant, μ is the oscillator’s reduced mass, ω_0_ its fundamental frequency, and *n* can be any non-negative integer. For convenience, we have omitted the kinetic term in the Hamiltonian.

If the system is implicitly solvated in a heat bath, its Hamiltonian is changed due to coupling, and it may be modeled as an effective Hamiltonian of mean force (see Section 3). As the equilibrium temperature increases, we expect the system to become softer and the force constant (the square of the frequency) smaller. In the high-temperature limit, the force constant vanishes. This physical behavior may be modeled by a temperature-dependent frequency ω(*T*) = ω_0_*e*^–*αT*^ (with a positive constant α modeling the strength of the coupling between the specific system and the particular bath).^25^ This results in a perturbed potential given by
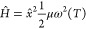
22

If the perturbed (nonisolated) system resembles the unperturbed (isolated) system, then the coupling constant α is small, and we may invoke first-order perturbation theory. This approximation is, of course, hardly necessary in this analytically tractable example, but it will serve its illustrative purpose.

The potential (22) has a zeroth-order contribution given by (20) and a first-order perturbation given by
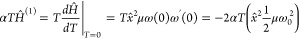
23The perturbed energy is given by (11), with *E*_*n*_^(0)^ given by (21) and *E*_*n*_^(1)^ by
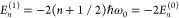
24This results in
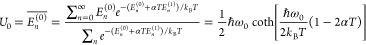
25
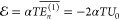
26
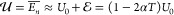
27For comparison, if the full Hamiltonian (22) were used, we would obtain  and .

This approximation is valid for sufficiently small values of the coupling constant α, which is the scenario where the system is well-described as a departure from a known isolated system, i.e., Hill’s fundamental approach in eqs 1 and (2).

When the system is very large (or fully isolated), then its eigenmodes are not perturbed by the thermal environment, and the coupling constant α → 0. In this scenario, , and the thermodynamic energy  reduces to the familiar result for the decoupled quantum harmonic oscillator (see, e.g., p 214 in ref (33)). However, in general, Hill’s interaction potential  and the energy . This will generally result in departures in pressure (15) and chemical potential (16) as described by Hill’s nanothermodynamic theory in expressions (3)–(5). These differences in pressure and chemical potential predicted by Hill result from strong interactions, and they have been shown to be the basis of thermodynamic laws valid at the nanoscale.^3^ Moreover, the temperature-dependent Hamiltonian of mean force (22) easily captures the somewhat surprising thermpophilic motion exhibited by some systems in solution.^25^

## 5 Concluding Remarks

Equilibrium statistical mechanics gives us a bridge between quantum mechanics and the continuous axioms of classical thermodynamics. One of these axioms is the additivity of extensive quantities. However, this property does not apply to small systems^5^ or generally to systems with sufficiently long-range interactions.^2^ Physical scientists usually accept that such systems are outside the scope of thermodynamic theory and simply move on.

This did not satisfy the prolific physical chemist Terrell L. Hill, who generalized standard Gibbsian thermodynamics to propose a purely thermodynamic framework for nonadditive systems.^4,5^ The general idea in Hill’s extended thermodynamic framework is fairly intuitive, and it facilitates the use of thermodynamics’ powerful tools in environments otherwise not accessible to classical thermodynamics (see e.g. refs (9−11, 34, and 35) and refs therein). However, Hill’s powerful framework does not reconcile with the standard thermostatistical treatment based on purely mechanical energy levels.

It is shown above that Hill’s theory may in fact be recovered by performing a standard thermostatistical treatment of energy levels if these are allowed to be effectively temperature-dependent (insofar as the Hamiltonian they result from may be perturbed by the heat bath). The presence of this perturbation allows us to invoke a temperature-dependent version of Rayleigh–Schrödinger perturbation theory and obtain a modified thermostatistical framework that produces Hill’s extension of classical thermodynamics, which in turn allows for a thermodynamic description of small nonadditive systems.
